# Sox2 enhances the tumorigenicity and chemoresistance of cancer stem-like cells derived from gastric cancer

**DOI:** 10.7555/JBR.26.20120045

**Published:** 2012-09-12

**Authors:** Tian Tian, Yajie Zhang, Shouyu Wang, Jianwei Zhou, Shan Xu

**Affiliations:** a Department of Cell Biology, Cancer Center, Nanjing Medical University, Nanjing, Jiangsu 210029, China;; b Department of Molecular Cell Biology & Toxicology, School of Public Health, Nanjing Medical University, Nanjing, Jiangsu 210029, China.

**Keywords:** side population, gastric cancer stem-like cells, CD44, Sox2, chemoresistance

## Abstract

Gastric cancer stem-like cells (GCSCs) have been identified to possess the ability of self-renewal and tumor initiation. However, the mechanisms involved remain largely unknown. Here, we isolated and characterized the GCSCs by side population (SP) sorting procedure and cultured sphere cells (SC) from human gastric cancer cell lines SGC-7901, BGC-823, MGC-803, HGC-27 and MKN-28. The sorting and culture assay revealed that SP cells proliferated in an asymmetric division manner. In addition, SP cells exhibited a higher potential of spheroid colony formation and greater drug resistance than non-SP cells (NSP). Moreover, the SC were found with enhanced capabilities of drug resistance in vitro and tumorigenicity in vivo. Sox2 mRNA and protein was highly and significantly overexpressed in the SP cells and SC. Importantly, downregulation of *Sox2* with siRNA obviously reduced spheroid colony formation and doxorubicin efflux, as well as increased apoptosis rate in sphere cells in vitro and suppressed tumorigenicity in vivo. These results suggest that both SP cells and cultured SC enrich with GCSCs and that Sox2 plays a pivotal role in sustaining stem cell properties and might be a potential target for gastric cancer therapy.

## INTRODUCTION

Gastric cancer (GC) is the second leading cause of cancer-related death worldwide[1]. Despite the development of surgery and chemotherapy, the 5-year survival rate across all stages is only about 20% due to metastasis, recurrence and multiple drug resistance (MDR).

Recent studies have revealed the critical role of cancer stem-like cells (CSCs) or tumor initiating cells in tumorigenicity and metastasis. CSCs are defined as a small side population (SP) of cancer cells, with the capability of both self-renewal and pluripotency[2], mediating tumor initiation, metastasis, recurrence and drug resistance[3],[4]. To date, the possible existence of CSCs has been identified in leukemia[5],[6], breast cancers[7],[8] and other solid malignancies[6],[9]-[11]. SP cells can be isolated by fluorescence activated cell sorter (FACS) that select cells exhibiting low fluorescence intensity of ABCG2-mediated efflux of Hoechst33342 dye, as CSCs are characterized by increased efflux potential, which results in reduced intracellular dye intensity[12],[13].

Recent evidence showed that cancers of the gastrointestinal system also contain SP cells displaying characteristics of stem cells[5],[14]. Gastric cancer stem-like cells (GCSCs) have recently been reported in GC cell lines and primary tumors[15] with the markers CD71–[16], EpCAM+/ CD44+[17] and CD90+[18] identifying them. These GCSCs possess a high migratory and invasive potential and enhanced drug resistance capability[16],[19]. However, the mechanisms involved remain largely unknown.

The *Sox2* gene encodes a member of the SRY-related HMG-box (Sox) family of transcription factors involved in the determination of cell fate and regulation of embryonic development and is a stem cell marker.

In this study, we demonstrated that SP cells isolated from CD44 high expression GC cell lines and cultured sphere cells (SC) displayed stem cell characteristics, correlated to elevated levels of ATP-binding cassette transporters (ABC transporters) and transcription factor Sox2. Importantly, siRNA-mediated silencing of Sox2 gene in SC remarkably suppressed tumor growth in nude mice gastric cancer xenograft. To the best of our knowledge, this is the first study to demonstrate that Sox2 is critical for sustaining the tumorigenicity of GCSCs.

## MATERIALS AND METHODS

### Cell culture and maintenance of tumor spheres

Five human GC cell lines, SGC-7901, BGC-823, MGC-803, HGC-27 and MKN-28 were purchased from the Institute of Biochemistry and Cell Research, China Life Science Academy (Shanghai, China). All the cells were cultured in RPMI 1640 (Gibco, Gaithersburg, MD, USA), supplemented with 10% FBS, 100 U/ml penicillin and 100 µg/ml streptomycin, at 37°C and 5% CO_2_. For spheres, SGC-7901 and BGC-823 cells were cultured in ultra-low-attachment 6 cm plates coated with poly-HEMA and with conditional GCSCs medium described as follows[7],[19].

### Spheroid colony formation assay

Both SP and non-SP (NSP) cells were sorted by FACS and then plated in ultra-low-attachment 96-well plates (Corning Life Sciences, Acton, MA, USA) at a density of 1 cell/well in GCSCs medium, containing DMEM/F12 (Invitrogen, Carlsbad, CA, USA) supplemented with human recombinant epidermal growth factor (EGF, 20 ng/mL), basic fibroblast growth factor (bFGF, 20 ng/mL) (PeproTech, Rocky Hill, NJ, China), B27-supplement (Invitrogen), insulin, 100 U/mL penicillin and 100 µg/mL streptomycin. After incubation for 2 weeks at 37°C and 5% CO_2_, colonies containing more than 50 cells were counted and the images were acquired with Image-pro Plus 6.2 software (Media Cybernetics, Bethesda, MD, USA) on IX70 inverted fluorescence microscopy (Olympus, Tokyo, Japan). The experiments were 96 paralleled (1 cell/well in 96-well plate) and repeated in triplicate.

### CD44, SP analysis and cell sorting

Procedures of SP analysis were performed as described in Haraguchi et al.[5]. We used FACS Aria Cell Sorter (BD Biosciences, Tokyo, Japan) for cell sorting and the data was analyzed by Flowjo software (Tree Star, San Carlos, CA, USA). For immunostaining, 1.0×10^6^ cells/mL single suspended cells were incubated with anti-CD44-FITC and isotype-specific IgG (eBioscience, San Diego, CA, USA) at 4°C for 20 min. Cells were then washed with PBS and analyzed on a Cytomics FC500 (Beckman-Coulter, Miami, FL, USA).

### Apoptosis assay

Briefly, 5.0×10^5^ cells were seeded onto 6-well plates, cultured in RPMI 1640 medium for 12 h, and then replenished with RPMI 1640 medium with or without 0.1 µg/mL doxorubicin and cisplatin for another 24 h. Next, cells were washed twice with PBS, and harvested by trypsinization and collected by 1500 rpm for 5 min. The cells were further incubated with 250 µL binding buffer and 3 µL Annexin V-PI incubation reagent (Bipec Biopharma, Cambridge, MA, USA) for 15 min before flow cytometric analysis. The experiments were performed at least three times and representative results were shown.

### Doxorubicin efflux analysis

For doxorubicin efflux analysis, cells were cultured in RPMI 1640 at 37°C for 60 min with or without 10 µg/mL doxorubicin and 100 µmol/L verapamil. After washing, the cells were released in drug-free medium for another 90 min and then subjected to flow cytometry assay to measure the intracellular intensity of doxorubicin fluorescence, which was excited by a 488 nm argon laser and assessed on fluorescence channel 2 at 575 nm wavelengths. The experiments were replicated at least 3 times and representative results were shown.

### Real-time RT-PCR

Procedures of RNA extraction and real-time reverse transcription PCR were as described by Hu *et al*.[20]. The primers we used are described in ***Table 1***. The experiments were done in triplicate.

**Table 1:** Real-time PCR primers

Gene	Primers
ABCG2	Primer1: 5′-TGGCTTAGACTCAAGCACAGC-3′
	Primer2: 5′- TGGTCCCTGCTTAGACATCC-3′
MRP1	Primer1: 5′-TGTGGGAAAACACATCTTTGA-3′
	Primer2: 5′-CTGTGCGTGACCAAGATCC-3′
MRP2	Primer1: 5′-AGTGAATGACATCTTCACGTTTG-3′
	Primer2: 5′-CTTGCAAAGGAGATCAGCAA-3′
MDR1	Primer1: 5′-ACAGAAAGCGAAGCAGTGGT-3′
	Primer2: 5′-ATGGTGGTCCGACCT TTTC-3′
CD44	Primer1: 5′-GCCTTGGCTTTGATTCTTGC-3′
	Primer2: 5′-TCCACTTGGCTTTCTGTCCTC-3′
HES1	Primer1: 5′-GAAAGATAGCTCGCGGCAT-3′
	Primer2: 5′'-GAAGCGGGTCACCTCGTT-3′
Krt15	Primer1: 5′-GCTGCTTGACATAAAGACACG-3′
	Primer2: 5′'-ATTGCTGCTGCTACCACCA-3′
Oct-4	Primer1: 5′-GGAAGGTATTCAGCCAAACGACCA-3′
	Primer2: 5′'-CTCACTCGGTTCTCGATACTGGTT-3′
Sox2	Primer1: 5′-TTCGATCCCAACTTTCCAT-3′
	Primer2: 5′'-ACATGGATTCTCGGCAGAC-3′
GAPDH	Primer1: 5′-GCCGGTGCTGAATATGTC-3′
	Primer2: 5′-CTTCTGGGTGGCAGTGAT-3′

### RNA interference

Sphere cells (5×10^5^) were seeded onto 60-mm plates coated with poly-HEMA and transfected with 10 µl of Sox2 siRNA mixture (sc-38408) or control siRNA (Santa Cruz Biotechnology, Santa Cruz, CA, USA), using Lipofectamine 2000 transfection reagent (Invitrogen). Forty-eight h after transfection, spheres were photographed and analyzed with Axiovision software on an Axiovert 200 M microscope (Carl Zeiss, Oberkochen, Germany). Spheres>100 µm were counted and cells were then washed once with PBS and harvested.

### Western blotting analysis

Total cell lysates were prepared using a detergent lysis buffer [50 mmol/L Tris (pH 7.4), 150 mmol/L NaCl, 1% NP-40, 0.5% sodium deoxycholate, 0.1% SDS, 1 mmol/L PMSF]. Western blotting performed as previously reported[21]. Three parallel samples were applied for each treatment group and 30 µg protein from the parallel samples were mixed and used for blots. The antibodies used were the polyclonal rabbit anti-Sox2 (Abcam, Cambridge, MA, USA), monoclonal mouse anti-ABCG2 (Abcam) and polyclonal anti-β-actin (Boster Biotechnology, Wuhan, China). Each blot was repeated three times.

### Xenograft assay in nude mice

To investigate the tumorigenicity of SGC-7901 and BGC-823 cells, we transfected sphere cells (SC) and adherent cells (AC) with Sox2 siRNA (si-Sox2) or negative control siRNA (si-con) for 48 h for xenograft assay. Six-week old female BALB/c nude mice weighing 18-22g were purchased from the SLAC Laboratory (Shanghai, China). All procedures involving the use of animals were approved by the Institutional Animal Care and Use Committee of Nanjing Medical University. The cells (2.5×10^5^) were resuspended in PBS and injected subcutaneously (*n* = 5). The tumor volumes were measured every 4 d and calculated based on the modified ellipsoidal formula: tumor volume=(length×width^2^)×0.5. At the end of 30 d, one mouse died in the group of mice injected with BGC-823-SC-si- con cells, the other mice were euthanized and the xenografts were removed and weighed.

### Statistical analysis

The statistical significance of difference between groups was performed using SPSS 15.0 software (SPSS, Chicago, IL, USA). Significant differences were determined by either Student's *t*-tests or one-way analysis of variance (ANOVA) tests followed by Student-Newman-Keuls tests and *P*-values < 0.05 were considered significant.

## RESULTS

### Isolation and characterization of CSCs from human gastric cancer cell lines

**Fig. 1:**
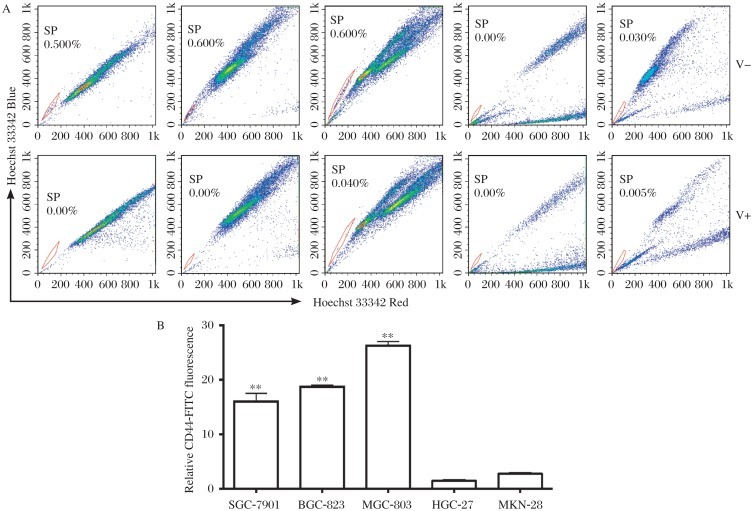
The side population (SP) analysis and CD44 expression of human gastric cancer cell lines. A: Representative SP analysis of five gastric cancer cell lines, the flow cytometry gate for SP cells was defined by the diminished area in the presence of 100 µmol/L verapamil. V: verapamil. B: Expression of CD44 in five human gastric cancer cell lines. SGC-7901, BGC-823 and MGC-803 cells expressed significantly higher CD44 levels than HGC-27 and MKN-28. Data was expressed as mean±SEM, **P < 0.01.

The SP cells were isolated from three out of the five cell lines (SGC-7901, BGC-823 and MGC-803) ***(Fig. 1A)***. The ratios of SP cells were 0.5% for SGC-7901 cells and 0.6% for both BGC-823 and MGC-803 cells, respectively. The SP cells completely disappeared from GC cells when treated with verapamil, an ABC-transporter inhibitor. It was disclosed that CD44 expression was significantly higher in SGC-7901, BGC-823 and MGC-803 cells than HGC-27 and MKN-28 cells (***Fig.1B***, F_(4,25)_ = 86.192, **P < 0.01). Both SGC-7901 and BGC-823 cells were then used in subsequent experiments.

**Fig. 2:**
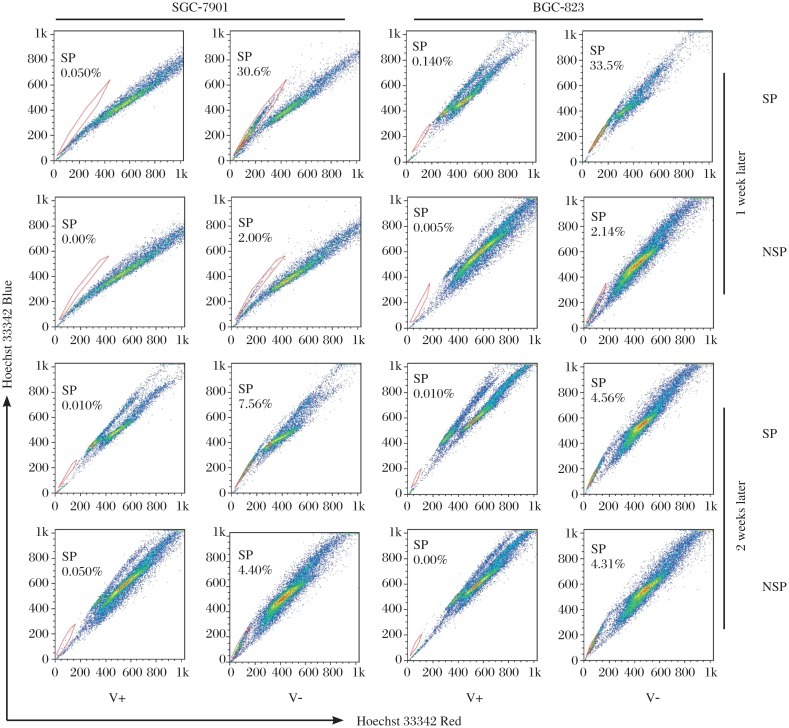
Asymmetric cell division-like proliferation of the SP cells. SP and NSP cells of SGC-7901 and BGC-823 cells were sorted and cultured independently in RPMI 1640 for 7 d and 14 d; and then evaluated for the appearance of SP cells. The ratios of SP fraction in cultured SGC-7901 and BGC-823 SP cells decreased from 30.6%, 33.5% to 7.56%, 4.56%, respectively, suggesting that SP cells regenerated both NSP and SP cells. However, NSP cells only regenerated NSP cells. SP: side population; NSP: non-side population.

**Fig. 3:**
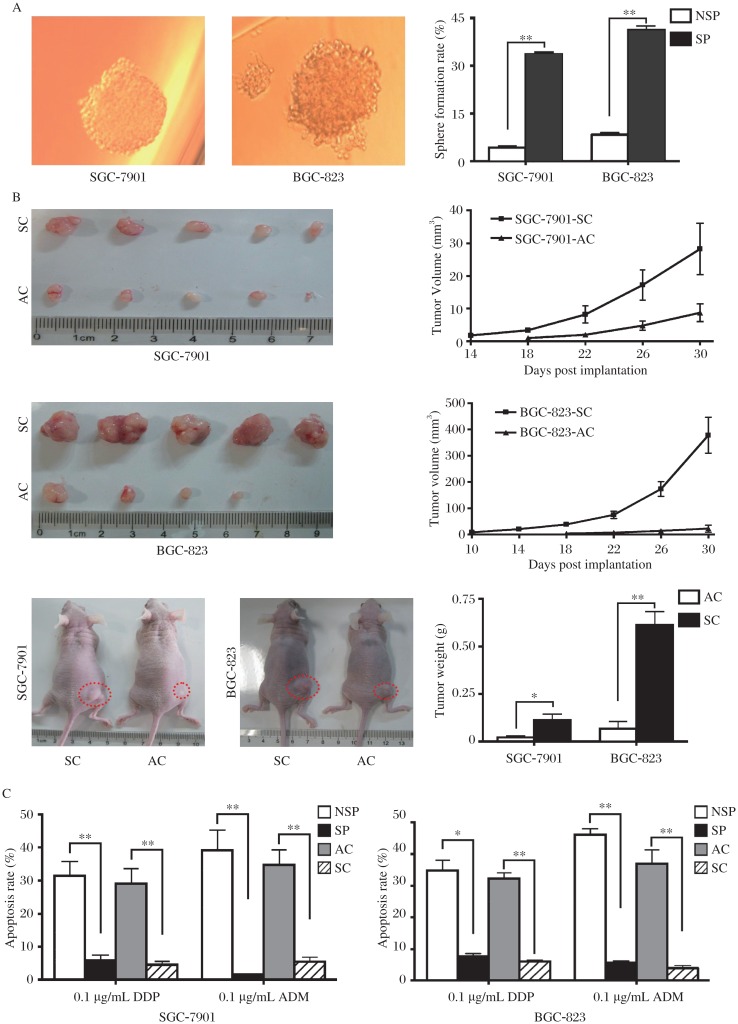
The SP cells and SC exhibit more stem cell like characteristics than NSP cells and AC. A: Potential of spheroid colony formation in SGC-7901 and BGC-823 cells was measured and SP cells generated more colonies than NSP cells. B: The tumorigenicity of SC and AC in SGC-7901 and BGC-823 cells was determined by xenograft assay in nude mice. SC formed more and larger xenografts than AC. C: SP cells and SC treatment with 0.1 µg/ml doxorubicin or cisplatin for 24 h induced lower apoptosis rate than NSP cells and AC. Data was expressed as mean±SEM, *P < 0.05, **P < 0.01. AC: adherent cells, SC: sphere cells, SP: side population, NSP: non-SP.

To verify if SP cells conferred asymmetric cell division, we evaluated post sorting SP and NSP fractions of SGC-7901 and BGC-823 cultured in RPMI 1640 medium for 7 and 14 d. The data indicated that SP fractions regenerated both NSP cells and SP cells, whereas NSP cells only regenerated NSP cells (***Fig. 2***). The spheroid colony-forming assay shown that SP cells formed more spheroid colonies than NSP cells (***Fig. 3A***, 8.4-, 5.0-fold, respectively, **P < 0.01), suggesting that a hierarchy -like system might exist in GC cell lines and that SP cells had high pluripotency capability.

### Tumorigenicity and chemotherapy resistance comparison between SC and AC or SP and NSP

We then compared the tumorigenicity potential between SC and adherent cells (AC) of SGC-7901 and BGC-823 cells. The data indicated that the volume of SC-derived xenograft tumors was significantly larger than that of AC-derived tumors (***Fig. 3B***, 6.2-, 11.9- fold, respectively, *P < 0.05, **P < 0.01).

Next, we analysed the chemotherapy resistance capability of SP, NSP fractions, SC and AC to 0.1 µg/mL cisplatin (DDP) or 0.1 µg/mL adriamycin (ADM), respectively. The result showed that SP cells and SC were more resistant to both doxorubicin and cisplatin than NSP cells and AC, respectively (***Fig. 3C***, *P < 0.05, **P < 0.01).

### ABC transporters and stem cell marker expression in SP and SC cells

**Fig. 4:**
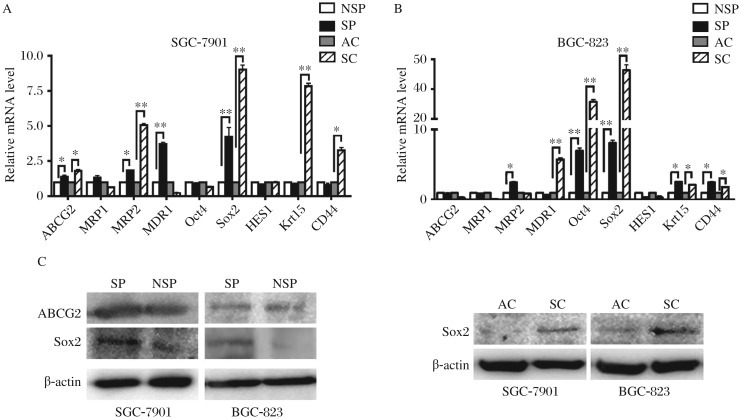
ABC transporters and stem cell markers expressed in gastric SP cells and SC. Q- RT-PCR (A, B) and Western blotting assays (C) were used to detect the expression of ABC transporters and stem cell markers in SP, NSP cells, AC and SC. Data was expressed as mean±SEM, *P < 0.05, **P < 0.01.

Overexpression of ABC transporters conferring active drug efflux is a well-known mechanism of MDR in several human cancers as well as markers of stemness. To determine if the intrinsic drug resistance of the gastric SP cells and SC might be related to elevated levels of ABC transporters, we conducted quantitative RT-PCR to identify the expression levels of such transporters (ABCG2, MRP1, MRP2 and MDR1) in these cells. Although the transcription factors and stemness genes *Oct4* and *Sox2*[22],[23], stem cell marker of the hair follicle bulge Krt15, the target genes of the Notch signaling pathway *HES1*[24] and *CD44*[6] were reported to be high expressed in some cancer stem-like cells, interestingly, in the present study, only *Sox2* was significantly overexpressed in SP versus NSP and in SC versus AC in both cell lines; while *Oct4*, *Krt15* and *CD44* were upregulated in one or both cell lines SP and/or SC (***Fig. 4A*** and ***4B***, *P < 0.05, **P < 0.01 ). As shown in ***Fig. 4C***, the results of Western blotting further confirmed that gastric SC and SP cells expressed markedly enhanced level of Sox2 protein than gastric AC and NSP cells. However, the ABCG2 protein was identical in both gastric SP and NSP cells. Taken together, we conclude that Sox2 may be essential in maintaining GCSCs properties.

### Sox2 is critical for gastric cancer stem-cell phenotype and tumorigenicity

**Fig. 5:**
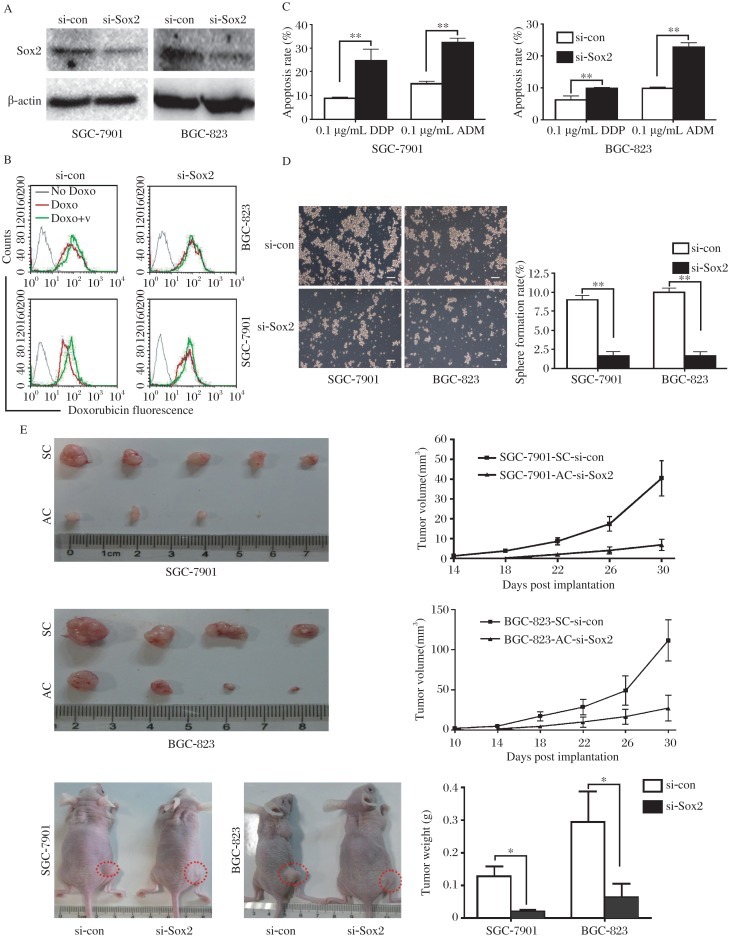
Knockdown of *Sox2* decreased GCSCs of GC cells. SGC-7901-SC and BGC- 823-SC were transfected with Sox2 siRNA (si-Sox2) or negative control siRNA (si-con) for 48 h. A: Western blotting was used to examine the expression of Sox2. Knockdown of Sox2 decreased the ability of doxorubicin efflux (B), increased apoptosis rate (C) and resulted in the low sphere formation (D) and tumorigenicity of SC (E). The doxorubicin efflux assay was done to show the activity of ABC transporters by the intracellular doxorubicin fluorescence difference between the verapamil-treated cells and the untreated cells. GC: Gastric cancer, AC: adherent cells, SC: sphere cells, SP: side population, NSP: non-SP. Data was expressed as mean±SEM, *P < 0.05, **P < 0.01.

To determine if Sox2 might play a vital role in sustaining the function of the GCSCs, single cell suspension of passaged SC was transfected with Sox2 siRNA or negative control for 48 h. The knockdown of Sox2 expression in SC of SGC-7901 and BGC-823 cells was confirmed by Western blotting (***Fig. 5A***). In vitro assays showed that silencing of Sox2 significantly decreased the ability of SC to expulse doxorubicin and form spheroid colonies (***Fig. 5B*** and ***5D***, 1/5.4-, 1/6.0- fold, respectively, **P < 0.01) and increased the apoptosis rate of SC when exposed to doxorubicin or cisplatin (***Fig. 5C***, **P < 0.01). Hereby, we demonstrate that Sox2 expression is directly linked to cisplatin and doxorubicin resistance in GC cells.

The tumorigenicity of *Sox2* knockdown SC in vivo was also addressed in nude mice. As shown in ***Fig. 5E***, compared with the control-siRNA cells, the growth speed and volume of tumors (1/5.7-, 1/3.7-fold, respectively, *P < 0.05) were profoundly reduced in mice injected with Sox2-siRNA SC cells.

## DISCUSSION

Important mechanisms in drug resistance include a greater capacity for DNA-damage repair, activation of survival and anti-apoptosis pathways as well as drug transport mechanisms. Chemotherapy often shows transient effects and hard to obviously improve patient prognosis. Even when therapies induce complete tumor regression, resistant sub-clones allow recurrence of the tumor. The CSCs are tumor sub-clones that display such characteristics. Here, we demonstrate that gastric SP cells and SC possess features of stemness and display an elevated intrinsic drug resistance, where overexpression of the transcription factor Sox2 and the drug transporter gene, *MDR1* and *MRP2*, may be involved. Moreover, a striking tumorigenic role of Sox2 was demonstrated.

Experimental evidence from the *Abcg2*-/- knockout mice model directly demonstrated that ABCG2 was the primary transporter mediating the SP phenotype and several other ABC transporters had overlapping function in Hoechst33342 dye efflux[25],[26]. Patrawala et al.[27] found that SP cells were enriched in tumorigenic CSCs, whereas ABCG2^+^ and ABCG2^–^ cancer cells were of similar tumorigenicity. In the present study, we found no significant change in protein levels of ABCG2 expression between gastric SP and NSP cells in both SGC-7901 and BGC-823 cells. Bleau et al.[10] and Hu et al.[20] demonstrated that the PI3K and Akt pathway was able to regulate the SP phenotype in human neurospheres, glioma and hepatocarcinoma cell lines via altering the subcellular localization of ABCG2 transporter, owing to its posttranslational modifications. Hence, in addition to ABCG2 expression level, the SP phenotype may be more relevant to the activity of ABCG2 transporter.

Apart from ABCG2, the overexpressed ABCA3 and MDR1 transporters have also been detected in SP cells[27]–[29]. Here, MDR1 was significantly overexpressed in SP and SC, and MRP2 was overexpressed in SP of both cell lines, indicating a role in chemoresistance of CSCs. Furthermore, MDR1 and MRP2 may be also associated with SP phenotype.

Sox2 plays a critical role in both neural stem cells and CSCs[30],[31] and may serve as a novel and potential biomarker for CSCs in gliomas. Interestingly, Gangemi et al.[32] investigated that Sox2-silenced glioblastoma tumor-initiating cells stopped proliferating and lost tumorigenicity. Sox2 expression was regulated by PLK1 in glioblastoma multiform cells and PLK1 inhibition could delay tumor progression in mice[33]. The Sox2 signaling pathway was essential in CSCs development and that its deregulation effectively suppressed growth and metastasis of non-small cell lung carcinoma cells[34]. Moreover, Sox2 may be related to gastric CSCs[35]. Clearly, the role of Sox2 in human tumors and specifically in GC is not clear as it was shown that loss of Sox2 expression may be related to gastric carcinogenesis and poor prognosis[36] while a recent study came to the opposite conclusion[37]. Here, we found that downregulation of Sox2 with siRNA reduced spheroid colony formation, and doxorubicin efflux and increased the apoptosis rate in GCSCs in vitro and significantly suppressed tumorigenicity in vivo.

In this study, for the first time, we have documented a high Sox2 expression in GCSCs and shown its pivotal role in chemotherapy resistance and tumor growth. Our data may help to develop more effective targeting therapy strategies in human GC.
